# Impact of Water Sources and Shared Fence Lines on Bovine Respiratory Disease Incidence in the First 45 Days on Feed

**DOI:** 10.3390/vetsci9110646

**Published:** 2022-11-21

**Authors:** Hector A. Rojas, Brad J. White, David E. Amrine, Robert L. Larson, Sarah F. Capik, Brandon E. Depenbusch

**Affiliations:** 1 Department of Clinical Sciences, College of Veterinary Medicine, Beef Cattle Institute, Kansas State University, Manhattan, KS 66506, USA; 2 Work Performed at Texas A&M AgriLife Research, Amarillo, TX 79106, USA; 3 Texas A&M School of Veterinary Medicine and Biomedical Sciences, College Station, TX 77843, USA; 4 Innovative Livestock Services, Great Bend, KS 67530, USA

**Keywords:** cattle, feedlot, bovine respiratory disease (BRD), water, fence line

## Abstract

**Simple Summary:**

Bovine respiratory disease (BRD) is a frequent disease in beef feedlot cattle, and little information is available on the role of pen housing conditions and risk for respiratory disease. The objective of this study was to evaluate potential associations between cohort (group) level respiratory disease risk with the number of water sources, if the water sources were shared between pens, and if the pen shared fence lines with other cattle. Results indicated that in certain instances (cattle weighing between 227–272 KG; group sizes of 100–175 head) having two water sources was associated with lower respiratory disease risk compared to only one water source. Shared fence lines and shared water sources did not have a biologically meaningful impact on respiratory disease risk. This research found that in specific situations the number of water sources was associated with respiratory disease risk.

**Abstract:**

Bovine respiratory disease (BRD) is a frequent disease in feedlot cattle, but little is known on the role of pen housing conditions. The objective of this research is to use a retrospective analysis with data from 10 U.S. feedlots to determine potential associations between BRD risk during the first 45 days after arrival with pen-level management factors including the number of water sources, shared water sources, and shared fence lines. Generalized linear mixed models were used to evaluate associations between management factors, cattle demographics, and BRD incidence. The effect of shared water sources on BRD risk was modified by arrival weight and cohort size (*p* < 0.05). Cattle with two water sources had lower BRD morbidity (5.55% ± 0.98) compared to cattle with one water source (8.80% ± 1.50) when arrival weight was 227 kg to 272 kg, while there were few differences in heavier weight cattle. Cattle with two water sources had lower BRD morbidity (3.11% ± 0.56) compared to one water (5.50% ± 0.10) when cohort size was 100–175 head, but there were no BRD morbidity differences when bigger or smaller cohorts were evaluated. Shared fence lines and water sources were associated with BRD risk; however, no biologically meaningful results were identified. The number of water sources was associated with BRD risk, and effects were modified by cohort size and arrival weight.

## 1. Introduction

Bovine respiratory disease (BRD) remains the primary cause of morbidity and mortality in feedlot cattle despite advancement in management and treatment protocols over the years [1,2,3]. Management and control of BRD is difficult as it is a multifactorial disease with several risk factors including arrival weight, time of year of arrival, commingling, and distance traveled to the feedyard contribute to onset of disease [4,5,6,7]. Knowledge gaps remain between the relationship of management interventions and health outcomes in feedlot cattle. The spread and transmission of pathogenic organisms in cattle populations has been documented as a risk factor towards BRD and multiple stressors such as dehydration and transportation may predispose cattle to viral infection and subsequent respiratory disease [8,9,10,11]. Understanding which interventions and management strategies are risk factors influencing BRD risk can be valuable towards mitigating BRD in feedlot cattle populations [12,13,14,15,16,17,18]. A previous study investigated housing factors related to shared pen water tanks and the number of adjacent pens and their associations towards BRD risk [19]. These studies reported associations between these risk factors and BRD incidence but did not look at the potential interactions between these management risk factors and other known risk factors related to cattle demographics on BRD risk. Previous work has illustrated that while pen management conditions such as bunk space per animal can be associated with BRD risk, these effects are not equal across all cattle demographic factors (arrival weight, cohort size) [20]. One study has illustrated that shared waters increased the risk of seropositivity to Mycoplasma bovis and thus a subsequent increased risk of BRD [21]. Evaluating the role of water sources and shared fence lines with BRD incidence may be useful for cattle managers, and the results should include the potential impacts of different cattle demographics on findings.

The objective of this study was to evaluate the potential associations between management characteristics, cattle demographics, and BRD incidence in the first 45 days on feed (DOF) in commercial feedlot cattle. The management factors evaluated are related to pen water sources and fence lines separating pens. Little work has been done with regard to evaluating the relevance of the number of shared waters or fence lines relative to BRD, and this information could be useful for future management decisions. Our goal was to find information regarding potential management interventions that would fill important knowledge gaps and enhance the understanding of management strategies that can be utilized by commercial feedlot operations to reduce BRD incidence.

## 2. Materials and Methods

Animal Care and Use Committee approval was not obtained for this study as data were collected retrospectively from commercial feedlots. Data were collected from 10 central U.S. high plains feedlots between January 2018 and April 2020 and included daily cohort- and individual-level information. Our outcome of interest was BRD incidence within the first 45 DOF as the majority of BRD onset occurs during this timeframe; therefore, all data were limited to events that occurred during the first 45 DOF. A cohort was defined as a group of cattle purchased and managed in a similar manner and housed together during the initial 45 DOF. Therefore, a cohort was a group of cattle arriving at a similar time and housed in the same pen the first 45 DOF. Cohort-level data consisted of demographic characteristics of the cattle including sex, arrival date, average arrival weight, and cohort size at arrival (Table 1). Individual animal data represented information on first treatment events for BRD and were joined to cohort level data using the yard, cohort ID, and pen ID where the treatment event occurred.

A variable representing a cohort-pen ID combination was created to track cohorts and their physical housing location (pen) during the 45 DOF period. Cohorts included in the study were restricted to those housed in two or fewer pens within the first 45 DOF and where transfer to the second pen occurred at less than 7 DOF. This cutoff was chosen as some cattle would be housed in a temporary pen for up to 7 days prior to moving to their final pen for the feeding phase. The pen characteristics utilized for the analysis were from the pen where cattle spent from at least day 7 to day 45. Any cohorts that were moved between three or more pens during the first 45 DOF were excluded from analysis. Individual animal data were filtered to only include animals with first treatment events for BRD within the first 45 DOF and were joined to their matching cohort.

Collected data were aligned with inclusion criteria, validated, categorized, and limited only to BRD-specific individual health events. Inclusion criteria were applied to remove categories with sparse data and criteria including removing cohorts with less than 25 animals, restricting average arrival weight between 227 kg to 453 kg, and including heifer, steer, and mixed-sex cohorts. Health related events not recorded as BRD were excluded from analysis (AIP, bloat, musculoskeletal, etc.). Data were collected from multiple feed yards and the BRD diagnosis was made by personnel in the field based on clinical signs of respiratory disease. The case definition for BRD diagnosis was based on individual criteria at each operation and may have varied slightly among personnel. Our case definition for BRD was the first time BRD was diagnosed in an individual animal by feedlot personnel and antimicrobial treatments administered during the first 45 DOF. Covariates were categorized to avoid violation of the linearity assumption similar to previous research [22]. Total cohort size at arrival (categorized as: 25 to 99, 100 to 175, >175) and average arrival weight (categorized as: 227 kg to 272 kg, 273 kg to 318 kg, 319 kg to 363 kg, 364 kg to 408 kg, 409 kg to 453 kg) were categorized similarly to previous literature [13,14]. Arrival dates were split into quartiles based on the arrival month to determine which quarter of the year the cohort entered the feedlot: January through March (Q1), April through June (Q2), July through September (Q3), and October through December (Q4). Cohorts with missing or incomplete data for any of these variables were excluded from the study population. The data came from a retrospective analysis from commercial feedlot data that consisted of information collected from multiple feedlot operations. As a result, vaccination programs from each operation, distance cattle traveled to the feedlot, and preconditioning status were unavailable.

Water sources placed in each pen were identified utilizing Google Earth Pro (version 7.3.3.7786) and the number of distinct water sources allocated per pen were counted. Cohort-level variables for water sources were created for each cohort which represented the number of water sources (NW) and shared water sources (SW). The definition for NW was the number of usable water sources available to cattle housed in each pen and was categorized into a binary variable representing having access to either one or two water sources per pen (no pens had >two water sources). The definition for SW was the number of water sources located in a pen that can be accessed by animals from one or more neighboring pens and was categorized into a binary variable representing no shared water sources or at least one shared water source. Any cohort that had missing water data was removed from the dataset for analysis.

A cohort-level variable for shared fence lines (SF) was assigned for each cohort and was defined as a fence line that is used to divide two separate pens from one another. The number of SF in each pen were documented utilizing Google Earth Pro (version 7.3.3.7786) and tallying the number of SF per pen. The variable for SF was categorized into a binary variable representing one shared fence line or two shared fence lines (no pens had greater than two shared fence lines). If a cohort had missing data for shared fence lines it was removed from the dataset for analysis.

Three distinct generalized linear mixed-models (NWmod, SWmod, and SFmod), one for each housing factor (NW, SW, and SF), were fitted with the “glmer” function in the ‘lme4’ package in R (R Core Team 2021) to assess potential associations between housing factors and cattle demographics with BRD incidence in the first 45 DOF. A logit link function was utilized in each model. The outcome variable of interest in each model was BRD incidence in the first 45 DOF and was calculated as the total number of first BRD treatments in the first 45 DOF (events)/total animal in the pen (trials). Covariates included average arrival weight, cohort size at arrival, arrival date quarter, sex, and one of the three housing factors. Several interaction terms were incorporated in each model based on previously investigated factors determined to affect BRD incidence in feedlot cattle including: sex with average arrival weight; sex with cohort size at arrival; sex with arrival date quarter; average arrival weight with cohort size at arrival; average arrival weight with arrival date quarter; and arrival date quarter with cohort size at arrival [3,15,16]. Interactions between housing factors and cattle demographics were included in each model. For example: the NWmod evaluating potential associations between NW and BRD incidence in the first 45 DOF tested the 2-way interactions between each cattle demographic and NW. A random intercept for feedlot was included in each model to account for the hierarchical structure of the data. Variables that were determined a priori to be associated with BRD based on previous literature (quarter of arrival, arrival weight, sex, animal received) were retained in the model as fixed effects regardless of statistical significance. Remaining variables (including interactions) were retained only if they were significantly associated (*p* < 0.05) with the outcome or were part of a significant interaction term. All main effects were included regardless of significance if they were part of a significant (*p* < 0.05) interaction.

## 3. Results

**Figure 1:**
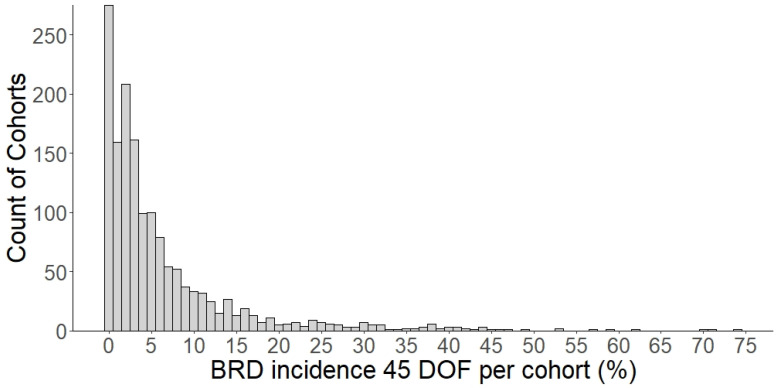
Histogram of the distribution of the level of BRD incidence in the first 45 days on feed (DOF) in the study population. The *x*-axis displays the percentage of BRD incidence in a cohort during the first 45 DOF. The *y*-axis displays the count of cohorts (pens) from 10 United States feedlots for each value.

Our study population included 1563 cohorts representing 168,482 individual animals from 10 feedlots. (Table 2). There were 10,065 recorded cases of first treatment BRD within the initial 45 DOF representing 6.44% of the study population. Figure 1 displays the distribution of BRD incidence in the first 45 DOF in the study population.

Table 3 lists all variables and interactions that were significantly associated (*p* < 0.05) with BRD incidence in the first 45 DOF in all models. All final models (NWMod, SWMod, SFMod) included the main effects of sex, cohort size at arrival, average arrival weight, and arrival quarter as-well-as significant (*p* < 0.05) two-way interactions between sex and average arrival weight, and average arrival weight and arrival quarter. In NWMod and SFMod, 2-way interactions between cattle demographics and the respective covariate of interest (NW or SF) were all significantly (*p* < 0.05) associated with BRD incidence. In the SWmod, the only significant (*p* < 0.05) interaction between the cattle demographics and the covariate of interest (SW) was cohort size at arrival and SW.

**Figure 2:**
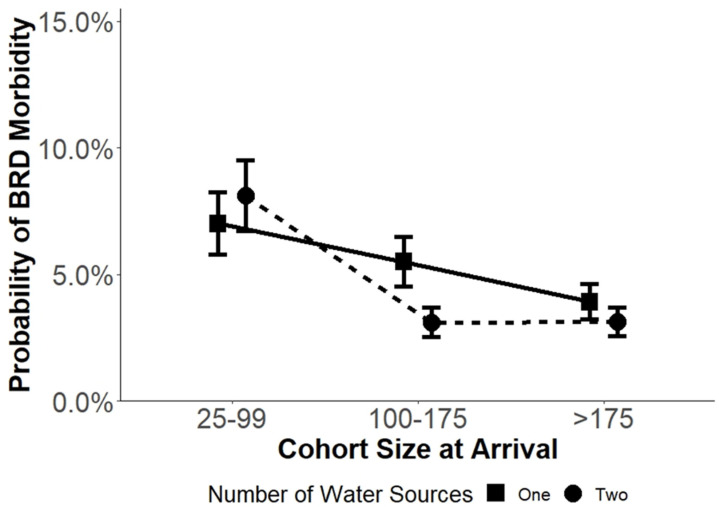
Model estimated mean probability of BRD incidence by total water sources and cohort size at arrival category in commercial feedlot cattle from 10 United States feedlots during the first 45 DOF using the NWmod *. Error bars represent SE of the probability. * NWmod is a generalized mixed model evaluating the number of water sources while including covariates for cohort sex, size at arrival, average arrival weight, arrival quarter and significant interactions.

The cohort size at arrival modified the effect of NW on BRD incidence in the first 45 DOF (Figure 2). There was a significant contrast seen in cohorts with a cohort size at arrival of 100 to 175 animals as cohorts in this category had a higher probability of BRD incidence in the first 45 DOF when provided with one water source (5.50% ± 0.10) compared to cohorts that had two water sources (3.11% ± 0.56). Cohorts in other categories of cohort size at arrival (25 to 99, >175) had similar probabilities of BRD incidence regardless of the total number of water sources available.

**Figure 3:**
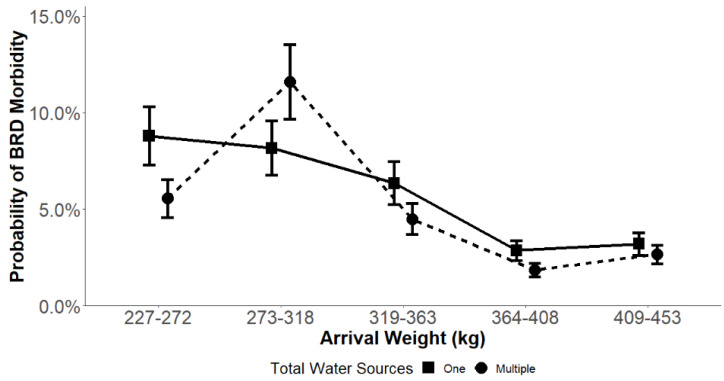
Model estimated mean probability of BRD incidence by total water sources and average arrival weight category in commercial feedlot cattle from 10 United States feedlots during the first 45 DOF using the NWmod *. Error bars represent SE of the probability. * NWmod is a generalized mixed model evaluating the number of water sources while including covariates for cohort sex, size at arrival, average arrival weight, arrival quarter and significant interactions.

The average arrival weight modified the effect of NW on BRD incidence in the first 45 DOF (Figure 3). There was a significant contrast seen in cohorts with an arrival weight between 227 kg to 272 kg in our results. In this average arrival weight category, the probability of BRD incidence was higher when cattle had one water source (8.80% ± 1.50) compared to cattle that had two water sources available (5.55% ± 0.98). There was also a significant contrast seen in cohorts with an arrival weight between 273 kg to 218 kg as the probability of BRD incidence was lower when cattle had one water source (8.17% ± 1.40) compared to cattle that had two water sources (11.60% ± 1.92). Cohorts in other average arrival weight categories did not display statistically significant differences in the probability of BRD incidence regardless of NW available.

## 4. Discussion

This study was conducted to evaluate potential relationships between feedlot management factors related to water sources/shared fence lines and cohort-level probability of BRD incidence within the first 45 DOF. Quantifying the effects of this relationship is important to determine the potential associations of potential cohort-level management factors and whether modifying these conditions could be used to mitigate BRD risk in feedlot cattle.

Prior research has investigated the relationship of these factors with BRD incidence in Australian feedlots [19]. However, our study evaluated three separate models incorporating interactions between housing factors and cattle demographics to assess if cattle demographics modified the effect of housing factors on BRD incidence in the first 45 DOF. Hay et al. (2017), reported that cattle with shared water sources were 4.3 times as likely (OR = 4.3, 95% CI = (1.4 to 10.3)) to be treated for BRD compared to cattle without shared water sources. In our study, the SWmod displayed a significant association between SW and the cohort size at arrival. Although this interaction was statically significant, it was not found to have biological significance. There were very few differences based on SW within cohort size for the arrival group and no meaningful trends were noted as the cohort size of the arrival group increased.

Hay et al. (2017) reported that the risk of BRD was not different between cattle that shared one fence line and cattle that shared two fence lines. In our study, the SFMod displayed that SF, as well as the interactions between SF and all cattle demographics, were significantly (*p* < 0.05) associated with BRD incidence; however, there were no biologically meaningful effects on BRD incidence. As a result, the current study and previous work [19] provided no evidence of meaningful associations between shared fence lines and BRD incidence.

The number of animals in a cohort has been associated with cohort-level risk of BRD [23,24,25,26]. In our study we observed a greater probability of BRD incidence within the 100 to 175 cohort size at arrival category when there was one total water source available compared to two water sources available. There were no significant differences in the probability of BRD incidence between one or two water sources within the 25 to 99 and >175 cohort size at arrival categories. Cattle management protocols may have influenced our results with pen selection based on the expected BRD risk of the cattle. Feedlot managers may be managing animals in this cohort size differently depending on the expected BRD risk of cattle. Commingling has been documented as a risk factor that affects the risk of BRD, and it can be inferred that the risk of BRD in different cohort sizes could be due to increased commingling in different sized groups [5,27]. Commingling status was not available in the current data; therefore, the potential impact of commingling could not be directly evaluated.

In the NWmod the probability of BRD incidence was similar between the 227 kg to 272 kg and 273 kg to 218 kg arrival weight categories, which were our lowest arrival weight categories. These findings are consistent with previous research that reported that light weight cattle are at higher risk for BRD compared to heavier cattle [4,22,28]. In addition, in the NWmod the probability of BRD incidence was lowest in the heavier weight arrival weight categories (364 kg to 408 kg and 409 kg to 453 kg). Within the 227 kg to 272 kg categories the probability of BRD was higher for cohorts that had one total water source available compared to cohorts that had more than one water. Changes in cattle feeding and watering behaviors have previously been associated with increased BRD risk [29,30]. The number of waters in the pen in newly arrived cattle may have had some influence on potential overall risk. In contrast, within the 273 kg to 218 kg category, cohorts with one total water source had a lower probability for BRD compared to cohorts with more than one water source. Management decisions regarding the physical placement of cattle within the feedyard based on BRD risk could confound our findings; however, this is not possible to evaluate based on our available data. In the SWmod the interaction between average arrival weight and SW was not statistically significantly associated with BRD incidence in the first 45 DOF. In the SFmod the interaction between average arrival weight and SF was statistically significant but did not display a biologically significant association with BRD in the first 45 DOF.

One limitation of our study is that it is a retrospective analysis looking at pre-existing observational data and may be subject to confounding or bias [31,32]. The results are also limited to feedlots that were included in the datasets utilized and may not be applicable to other feedlots due to differences in management, dates of data recorded, geography, cattle types, different case definitions for BRD, and many other potential differences. Because our data originated from commercial feedlots, it is inherently “messy” and may contain unknown biases or errors despite our efforts to clean and validate it. Additionally, we only evaluated BRD incidence during the first 45 DOF, which did not encompass all total BRD treatments throughout the entire feeding phase. It is possible that some of the risk factors we explored related to BRD incidence are significantly associated with risk of retreatment, risk of becoming a chronic animal, or risk of dying; our analysis did not include those outcomes due to limitations of the data available. Additional studies will be needed to evaluate those other important outcomes and their relationship with management-related risk factors. Several cohorts were also removed from the dataset if they were housed in more than 2 pens throughout the first 45 DOF. There are many possible reasons related to cattle flow and management decisions that may have caused cohorts to move several times throughout the first 45 DOF. As the objective of this study was to evaluate potential associations between pen waters and shared fence lines, it would become extremely difficult (if not impossible) to evaluate these characteristics from more than one pen and their potential associations with a single value of BRD incidence within the first 45 DOF. Finally, the risk status of cohorts entering the feedlot was not known in our dataset, so we could not determine which cohorts of cattle were classified as high risk for BRD at arrival. A future, well-controlled prospective study examining the risk of BRD during the first 45 DOF in association with pen housing conditions that includes some of the missing metadata should be conducted to help determine the differences in BRD incidence.

## 5. Conclusions

Our study determined that the probability of BRD incidence in the first 45 DOF was significantly affected by interactions between management variables related to water sources and shared fence lines. However, these interactions did not display biologically meaningful differences that can be used to better manage BRD incidence in feedlot cattle. These management variables and their impact on BRD incidence have been briefly evaluated in previous literature, and our study further elaborates on the associations of those variables on BRD incidence early in the feeding period and how they are affected by other well-known cattle demographic risk factors. Results from this study provide estimates on how these management factors may be influencing the risk of initial BRD treatment for feedlot cattle. Additional research in this area will lead to a better understanding of the effects of management conditions for feedlot cattle and how these conditions can potentially be modified to reduce the risk of BRD in the feedlot industry.

## Figures and Tables

**Table 1:** Distribution of variables used for analysis from 10 United States feedlots from January 2018 to April 2020.

Variable & Category	Number (%) of Cohorts
Cohort size at arrival	
25–99	854 (55.74)
100–175	447 (29.18)
>175	231 (15.10)
Average Arrival Weight, kg.	
227–272	116 (7.57)
273–318	317 (20.69)
319–363	558 (36.23)
364–408	407 (26.57)
409–453	134 (8.74)
Sex	
Heifers	819 (53.46)
Steers	599 (39.09)
Mixed	114 (7.44)
Arrival Date Quarter	
Jan–March (1)	398 (25.97)
April–June (2)	433 (28.26)
July–September (3)	419 (27.34)
October–December (4)	282 (18.40)
Total Water Sources	
One source (0)	1342 (87.60)
Multiple sources (1)	190 (12.40)
Shared Pen Waters	
No (0)	531 (31.10)
Yes (1)	1001 (68.9)
Shared Fence Lines	
One (0)	428 (27.94)
Two (1)	1104 (72.06)

**Table 2:** Distribution of variables used for analysis from 10 United States feedlots from 2018–2020.

Variable	Mean	SD ^2^	Median	Range	IQR ^3^
Cohort Size at arrival	108.69	55.88	87	25–324	64–144
Average arrival weight, kg	345.2	102.9	346	228–453	314–378
BRD incidence ^1^, %	6.44	9.00	3.26	0–74.07	1.28–7.84

^1^ First treatment bovine respiratory disease (BRD) incidence was our outcome variable and was calculated only for the initial 45 days on feed. ^2^ SD = standard deviation. ^3^ IQR = interquartile range.

**Table 3:** Final generalized linear mixed-models demonstrating housing characteristics and cattle demographic factors and their association with bovine respiratory disease incidence during the first 45 DOF. Data were included from 10 United States feedlots from 2018–2020. Three models (NWmod, SWmod, SFmod) for each hosing factor (number of waters, shared waters, and shared fence lines) were evaluated.

Variable	NWmod *p*-Values	SWmod *p*-Values	SFmod *p*-Values
Sex	<0.01	<0.01	<0.01
Cohort size at arrival	<0.01	<0.01	<0.01
Average arrival weight	<0.01	<0.01	<0.01
Arrival date quarter	<0.01	<0.01	<0.01
NW ^1^	<0.01	--- ^4^	---
SW ^2^	---	0.27	---
SF ^3^	---	---	<0.01
Sex x Average arrival weight	<0.01	<0.01	<0.01
Average arrival weight × Arrival date quarter	<0.01	<0.01	<0.01
Sex × NW	<0.01	---	---
Average arrival weight × NW	<0.01	---	---
Cohort size at arrival NW	<0.01	---	---
Arrival date quarter × NW	<0.01	---	---
Sex × SW	---	0.77	---
Average arrival weight × SW	---	0.07	---
Cohort size at arrival SW	---	<0.01	---
Arrival Date Quarter × SW	---	0.63	---
Sex × SF	---	---	<0.01
Average arrival weight × SF	---	---	<0.01
Cohort size at arrival × SF	---	---	<0.01
Arrival Date Quarter × SF	---	---	<0.01

^1^ NW = number of water sources. ^2^ SW = shared water sources. ^3^ SF = shared fence lines. ^4^ Dashed lines (---) signify that the variable was not incorporated into the model.

## Data Availability

Data utilized for this research were from cooperating entities and are not available publicly due to confidentiality and anonymity agreements.
